# The Importance of Demonstratively Restoring Order

**DOI:** 10.1371/journal.pone.0065137

**Published:** 2013-06-05

**Authors:** Kees Keizer, Siegwart Lindenberg, Linda Steg

**Affiliations:** Faculty of Behavioral and Social Sciences, University of Groningen, The Netherlands; Northwestern University, United States of America

## Abstract

Contrary to what is often assumed, order is not the strongest context for encouraging normative behavior. The strongest context effect on normative behavior comes from cues that clearly convey other people’s respect for norms. Ironically, this show of respect necessitates some contrasting disrespect that is being restored. Using civic virtues (such as helping behavior) as a prototype of normative behavior, the three field experiments described in this paper reveal the impact of normative cues on civic virtues. Results show that the strongest effect on making people follow prosocial norms in public places emanates from seeing order being restored, rather than just order being present. The robust and surprisingly large effects show that observing other people’s respect for one particular norm (as evidenced in their restoring physical order) makes it more likely that the onlooker follows other norms as well. This implies that prosocial behavior has the highest chance of spreading when people observe order being restored. There are clear policy implications: create low cost “normative respect cues” wherever it is desirable to increase conformity to norms.

## Introduction

Disorder spreads. For example, littering is a widespread phenomenon [1] and, as it is a sign of physical disorder, it encourages more littering [2] but also other transgressions of rules and norms like stealing [3]. These effects are surprisingly strong and paint a gloomy picture of spreading negative behavior triggered by cues of physical disorder. Might the picture actually be less bleak? Can socially positive behavior, that has recently received much attention for its important contribution to quality of life [4]–[7], spread as well? If so, how might prosocial behavior come to spread?

An explanation for the spreading of disorder that seems popular with policymakers is that people in such settings reason (based on the signs of norm violating behavior by others) that they will not get sanctioned for their own transgressions. [8]. The focus theory of norms [2] argues that people imitate norm-breaking behavior of others with the assumption that seemingly this is the way to do it, if others are doing it. Both of these assumptions do not explain, how it is possible that seeing norm A being violated, people are more likely to transgress norm B as well. This has been called the “cross-norm effect” [3]. Goal framing theory [9] suggests that this cross-norm effect works on the basis of overarching goals. It predicts that the relative weight of overarching goals (such as the goal to behave appropriately – i.e. to keep to social norms- versus the goal to feel good and thus not exert effort if it is not rewarded) is affected by cues about the behavior of others. Although complying to rules is the appropriate behavior, transgressing them is often less effortful or more profitable. In other words, people experience a conflict of goals. Goal framing theory suggests that in such cases, the likelihood of acting according to social norms strongly depends on cues that strengthen or weaken the goal to act normatively [10]. Other people’s explicit behavior or behavior that is reflected in visible order or disorder in the environment is crucial in this respect. According to this view signs of disorder like litter or graffiti might be indications for the probability of getting sanctioned; but even if they are not, they are cues that signal a lack of other people’s support for norms. Such *normative disrespect cues* (i.e. “disrespect cues”) induce rule transgressions by weakening people’s goal to act according to norms. This reasoning has a series of important implications. First it suggests that disrespect cues will also negatively influence the likelihood of complying with norms in situations where the sanction probability is clearly low for other reasons than disorder (e.g. when people are unobserved). Second, it predicts that the inhibiting effect of disrespect cues applies also to prosocial behavior like being helpful towards strangers. Conforming to such norms takes effort and time and thus creates a conflict of goals, but there is typically no association with sanctions for transgression. Our first hypothesis thus is that acting prosocially is inhibited by disrespect cues. Our second, and most important hypothesis regarding the spreading of prosocial behavior, derives from a third implication of Goal framing theory about *normative respect cues* (i.e. “respect cues”). According to the theory, the absence of disorder (i.e. order) is itself a subtle cue of normative respect of others. However, explicit display of respect for norms is expected to be a much stronger respect cue and should therefore have an even stronger effect on the relative strength of the goal to follow norms. Our second hypothesis is thus that the likelihood of prosocial behavior will increase when the intensity of a respect cue increases. The existence of such a “graduated cross-norm reinforcement” effect in the positive range of respect for norms (rather than just lack of disrespect) would imply that display of explicit prosocial behavior has a high chance of spreading. If true, the graduated cross-norm effects could provide a new perspective on normative behavior in public places.

We tested these expectations in a series of three (between subjects) field experiments on helpfulness in the public realm. In these studies we gradually increased respect for norms signaled by simple normative cues: from subtle normative disrespect cues to intense normative respect cues.

## Experiments

**Figure 1:**
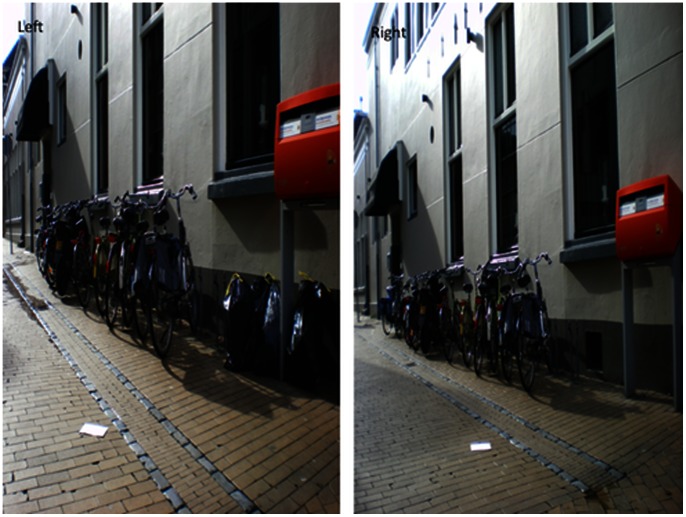
Setup in Study 1. **Left**, Photograph of the setup of in the “Garbage bag” condition. **Right**, Photograph of the setup of in the “Clean” condition.

**Figure 2:**
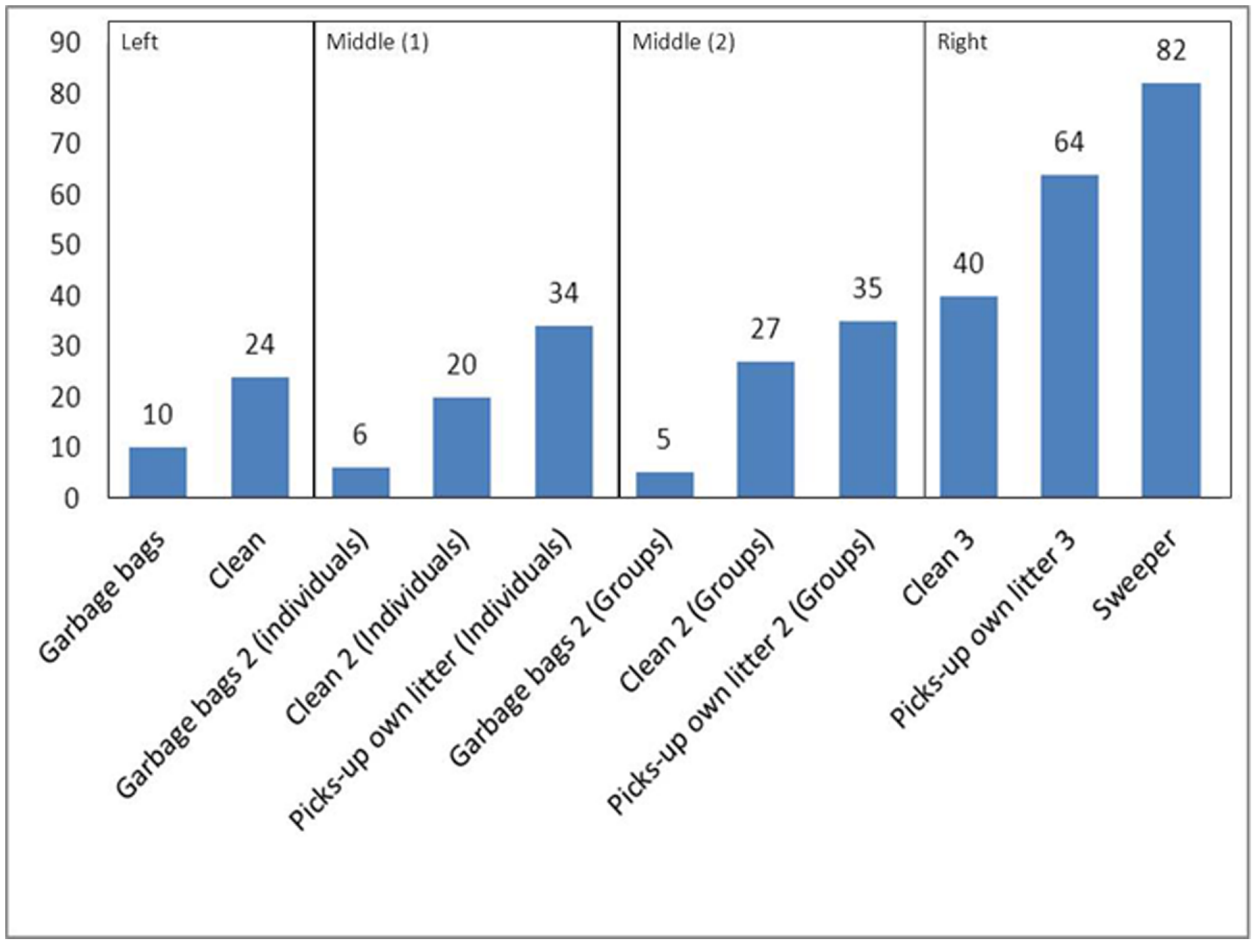
Effect of intensity of normative (dis)respect cues on prosocial behavior. Percentage of participants in the different conditions in the three studies that acted prosocially. **Left**, Study 1: Percentage of participants that posted the lost letter. **Middle (1)**, Study 2: Percentage of individual participants that picked up the fallen bicycle. **Middle (2)**, Study 2: Percentage of groups that picked up the fallen bicycle. **Right**, Study 3: Percentage of participants that helped by picking up the dropped oranges.

In our first field experiment we examined whether disrespect cues indeed inhibit prosocial behavior (Hypothesis 1). We investigated which percentage of the passersby (i.e. passing individuals or groups) acted prosocially by posting an envelope that apparently had fallen from a nearby letterbox (our dependent variable. A group of people (i.e. at least two persons seemingly belonging together) was regarded as a single observation. In our first condition (See Figure 1 Left) we placed a couple of garbage bags (a subtle disrespect cue) in the setting. In the city in which the experiments were conducted (Groningen, the Netherlands), it is not allowed to place garbage bags in the street. In this condition (See Figure 1 Right) (*N* = 147) 10% of the passerby stopped to pick up and post the letter. However, when we removed all signs of disorder (representing a very subtle respect cue, *N* = 136), the percentage of passersby that helped was 24%. The increase in prosocial behavior between the subtle disrespect and subtle respect condition (see Figure 2 Left) is support for our hypothesis that prosocial behavior is decreased by disrespect cues (*z* = 3.149, *p* = .001 one-sided).

A correlational study by Nettle et al. [11] which also used a lost letter approach for measuring prosocial behavior showed that letters dropped in more littered and criminal neighborhood were less likely to be returned. The present work supports the idea that such effects are not necessarily caused by socioeconomic differences but can also stem from differences in the presence of disrespect cues. Conversely, it shows that prosocial behavior is more likely when the absence of disrespect cues subtly signals respect for social norms.

**Figure 3:**
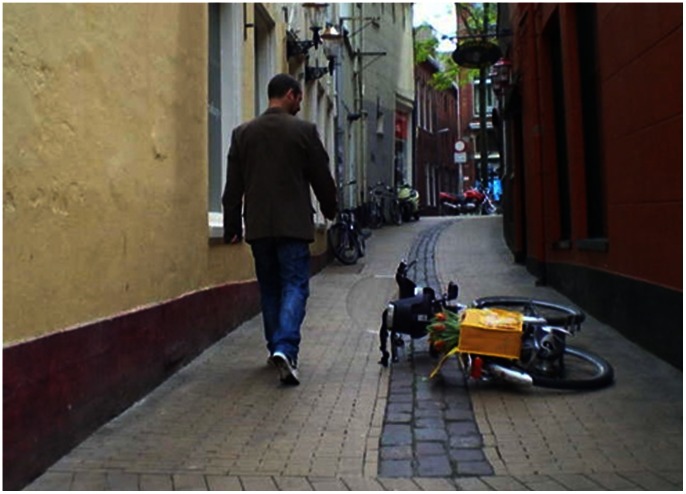
Photograph of the setup in the in the “clean” condition of Study 2.

For the spreading of prosocial behavior it is especially important to see whether there is a graduated cross-norm reinforcement effect. On the basis of Goal framing theory we would expect a more intense respect cue (i.e. a cue that conveys more than the mere absence of disrespect) to further increase the likelihood of prosocial behavior (Hypothesis 2). To test this graduated cross-norm reinforcement effect, we designed another field experiment and we added two additional questions. First, in our previous study observations consisted of both individuals and groups. This left us with the question whether individuals and groups differ in their reaction to normative cues. To answer this question we decided to look at groups and individuals separately. Second, in Study 1, the cost of acting prosocially was very low. Would normative cues also increase prosocial behavior when it takes more effort than picking up a letter? To address these questions, we investigated whether people would pick up a bicycle that apparently had accidentally fallen over (see Figure 3). In this study, we had three conditions: a subtle disrespect cue, a subtle respect cue that (merely) conveyed lack of disrespect (i.e. order), and a respect cue that conveyed a moderate degree of explicit respect for norms. In the disrespect condition, we used again garbage bags as a subtle cue of normative disrespect. In the subtle respect (order) condition, the street was clean, and in the more intense (“moderately intense”) respect condition, passersby observed a confederate dropping an empty soda can *and picking it up again* (a clear sign of respect for the anti-litter norm) before they entered the alley and faced the fallen bicycle. In the disrespect condition, 6% of the individual passersby (*N* = 77) and 5% of the groups (*N* = 21) acted prosocially by putting a clearly accidentally fallen bicycle back on its standard. Similar to Study 1, the percentage of passersby acting prosocially more than doubled in our very subtle respect condition, i.e. when the alley was clear of disrespect cues. In this case, 20% of the individuals (*N* = 66) and 27% of the groups (*N* = 26) stopped to pick up the bicycle. However, in the more explicit, moderately intense respect condition, prosocial behavior was even more likely: 34% of the individuals (N = 56) and 35% of the groups (*N* = 43) stopped to pick up the bicycle. A polynomial contrast analysis for both individuals (*b* = 1.42, *Wald*(1) = 13.64, *p* = .000) and groups (*b* = 167, *Wald*(1) = 4.881, p = .027) supports our hypothesis that prosocial behavior becomes more likely as the observed respect for norms increases, (see also Figure 2 middle 1 and 2). No difference between groups and individuals was found in any of the conditions (Analysis S1) an indication that both were influenced (equally) by subtle normative cues.

**Figure 4:**
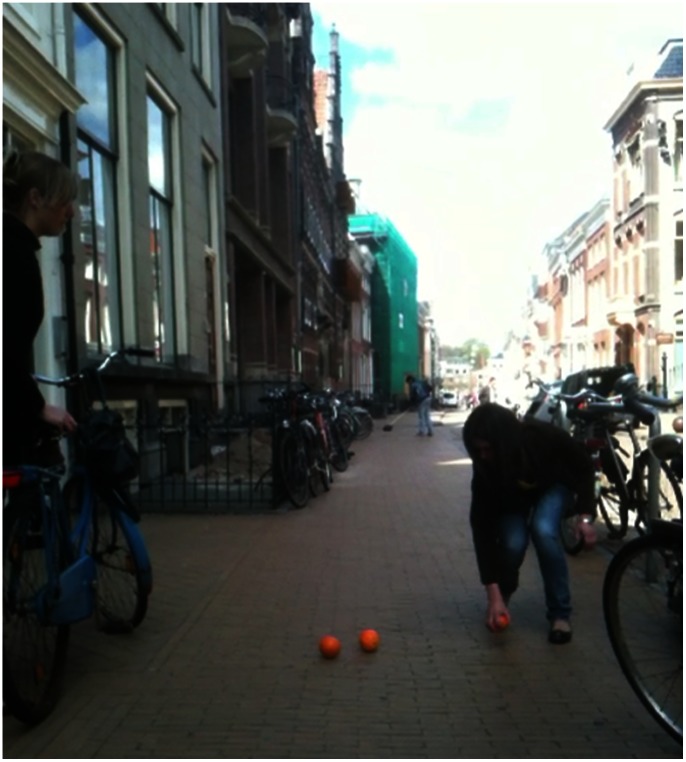
Photograph of the setup in the “sweeper” condition of Study 3.

On the basis of the graduated cross-norm reinforcement effect, we would expect that the percentage of people showing prosocial behavior could be pushed up even more with a more intense normative respect cue. To test this and to see whether the effect would hold not just in anonymous situations (such as picking up a letter or bicycle) but also in the mutual physical presence of potential helper and helped, we designed Study 3 (this time with only individuals, no groups). Here we observed whether individual passersby would help a confederate who “accidentally” dropped a number of oranges as they approached (see Figure 4). In our very subtle respect cue condition no signs of disorder were present. Before entering the experimental scene, participants passed our second confederate (who merely walked in the opposite direction as a human presence). In our moderately intense respect cue condition, the second confederate dropped an empty soda can *and picked it up again*, thereby clearly showing respect for the anti-litter norm. In our normative respect condition of highest intensity (see Figure 4), our second confederate (seemingly a resident) was restoring order by sweeping the street, visually and audibly removing not (just) his or her own litter but rather the results of transgressions by others. People passed this sweeper (and thus the intense respect cue) about 20 meters before they reached the confederate who dropped the oranges. The resulting percentages of prosocial behavior clearly followed the respect cue intensity: 40% the very subtle respect cue conditions (*N* = 50), 64% in the moderately intense respect cue condition (*N* = 56), and 82% in the intense respect cue condition (*N* = 61). A polynomial contrast analysis supports our hypothesis that as the respect cue intensifies (i.e. the observed respect for norms increases), prosocial behavior becomes more likely (Figure 2 right; *b* = 1.357, *Wald*(1) = 18.970, *p* = .000). Clearly, cues that show respect for norms have a positive effect on the observer’s readiness to behave prosocially, the more so, the more intense the cue of respect for norms.

## Discussion

The present studies show that the realization of civic virtues in the public space can be better achieved when there is not just a lack of disorder but clear signs of respect for order norms. Our results show that in the public space, the intensity of normative respect cues matters. It was already known that cues of disrespect lower people’s willingness to comply to norms that are typically associated with sanctions [3]. However, here we could show that, in addition, the same influence applies to norms that are not associated with sanctions when transgressed, such as the prosocial norm of helping others. More importantly, we could show that people react in a graduated manner to positive normative cues in the environment. The more respect conveyed by a cue the more prosocial behavior of the observer. In this sequence, “order” is a rather modest sign of respect for norms. There are stronger ones, namely cues conveying explicit care for norms. It is this graduated cross-norm reinforcement effect that can make prosocial behavior spread from one norm to another. The present findings not only leave us with this insight but also with a clear policy message. The “broken-window” theory kind of advice was: keep it clean [3], [8]. However, one can do better than that. In the public space, prosocial behavior is vital for quality of life and it does not seem to take expensive programs to boost it. One should demonstratively restore order, preferably with cues showing a high intensity of respect for norms. This would also hold for the way the police should operate [12]. A general piece of advice for cities implied by our findings is “don’t just keep it clean but clean up when people see it and display some effort in doing so”. Demonstratively showing respect for norms by simple cues can make a big difference. Recall that in the public space, one person armed with a broom was able to boost helping others in need by more than 100 percent.

## Methods

### Ethics Statement

The conducted studies were observational field experiments. Given the nature of the studies, informed consent was not possible. However participants were all people in public spaces (where displayed behaviour is generally visible to others). We also did not record any personal identifying information. The University of Groningen Psychology Ethics Committee waived the need for written informant consent from the participants. The “participants”, in figures 3 and 4 are played by confederates. This was done for privacy and practical reasons. All the people in figure 3 and 4 (i.e. all confederates) have given written informed consent, as outlined in the PLOS consent form, to publication of their photograph.

The field studies were all situated in Groningen, a town in the North of the Netherlands. All locations were in the city center in a public area with predominantly shoe and clothing shops. These locations were chosen to guarantee a cross section of the general population and to reduce the possibility of one participant reappearing in the same or different conditions (as would be more likely in case of a location near an apartment building or supermarket). All studies were run during the same part of the day (in the afternoon) and under the same weather conditions (no rain, partly cloudy). Participants in Studies 1 and 2 were individuals or groups of people (who obviously belonged together) that passed the experimental setting on foot. In Study 3 only individual pedestrians were investigated. In all studies, only individuals that were estimated to be over 18 year old where included or (when applicable) groups whose members were all estimated to be over 18. Present normative cues (e.g. graffiti or litter) other than the ones that were purposefully placed were removed prior to the experiment. Observations in all studies were done by an out-of-sight observer.

### Study 1

Participants were individuals or groups of people (who obviously belonged together) who were not followed by others within a range of approximately 12 meters. Participants who could observe another participant ignoring or posting the envelope were also not taken into account. The setting was part of a street that leads up to a shopping area. Although the street is located in a busy area, the experimental setting itself was relatively quiet and predominantly used by pedestrians (cars are not allowed in this area) going to and coming from a nearby car parking venue. Before the experiment, all signs of norm-violating behavior (like litter) were removed. A confederate waited until the setting was clear of people before (re)placing the envelope in its designated location. The experiment started (again) when the confederate had left the scene. The envelope used in the experiment was sealed (front facing upward), addressed and had a valid stamp. This was done to strengthen the impression that the envelope had fallen from the mailbox or accidentally dropped by someone.

To create the subtle disrespect condition we took a couple of garbage bags and placed these in the setting. The bags were positioned in such a manner that every participant looking at them would at least glance at the envelope. This was done to avoid that participants who looked at garbage bags would not see the envelope. We used garbage bags instead of litter to avoid that the envelope would be perceived as litter. In the entire inner city of Groningen, it is not allowed to place garbage bags on the street (they have to be brought to a special container).

### Study 2

Participants were either individuals or coherent groups walking towards a shopping area. The setting was an alley in the center of Groningen. Apart from an occasional cyclist, the alley is mainly used by pedestrians. People visiting nearby shops sometimes use the alley to park their bicycle. Before the experiment again all signs of norm violating behavior were removed. This implied removing some litter and painting some parts of the alley wall to remove a few tags. The bicycle was positioned in such a manner that participants could easily walk around it. This was done to make sure that picking up the bicycle was done for prosocial reasons and not for reasons of one’s own convenience. An out-of-sight confederate noted whether the bicycle was picked-up and whether the observation concerned a group or an individual. In case of an individual, the gender of the participant was recorded. No relationship between the gender of the participant and the likelihood of engaging in prosocial behavior was found in any of the conditions (Analysis S1).

In all conditions, participants faced the fallen bicycle approximately 5 meters after turning the corner to enter the alley. The experiment was temporarily stopped after a participant picked up the bicycle and started again after our confederate had placed the bicycle at the same location, in the exact same position and had left the scene.

The garbage bags in our first subtle disrespect condition were similar to the bags used in Study 1. They were placed in the alley, approximately 5 meters from the bicycle against the alley wall. They were positioned in such a way that participants looking at the bicycle would also see the garbage bags and vice versa.

The confederate in our moderately intense respect condition was walking towards potential participants, along the street where the alley entrance was located (i.e. the confederate did not enter nor exit the alley). When the potential participants had approached to about 10 meters, the confederate “accidentally” dropped an empty soda can and picked it up again. The sound of the can hitting the ground not only made very clear that the can was empty, but also drew attention to the act of dropping it and subsequently picking it up.

### Study 3

The setting was a sidewalk of about 4 meters wide. The participants were individuals walking towards the city center. Similar to Study 3 it was recorded whether a participant was a man or a woman but no gender differences were found in any of the conditions (Analysis S2). The “orange” confederate was standing far from the roadside between a building and a bicycle which (s)he held with a bag of ranges dangling from the handlebar. The sex of this confederate could have an influence on whether or not participants helped. One might for example reason that people would be more likely to help someone of the opposite sex. To account for these unwanted effects, both male and female confederates were used, support for such an interaction effect was only found in condition 1 (Analysis S2). We used oranges, as they were highly noticeable and would easily roll away. When dropped, the oranges rolled towards the street away from the confederate (i.e. the pavement was slightly sloping towards the street) thereby crossing the path of the approaching participant. After the confederate had “accidentally” dropped the oranges, he or she seemingly tried to retrieve them even though it was quite troublesome to do so because she had to put the bike on a stand and walk around the bicycle. He or she clearly tried to put the bicycle on its stand. This was done to generate the impression that the confederate was not expecting the participant to pick up the oranges. Also no eye contact was made with the passerby in order to make sure that no impression of a request for help would be generated. Our confederate was still busy putting the bicycle on its stand when the participant reached the location of the fallen oranges. We considered picking up at least one of the oranges as helping. Kicking an orange in the direction of the confederate (happened once) was not considered as helping behavior. After the scene had taken place, the experiment was temporarily stopped until the starting position was taken again and all people that could have witnessed the scene had left the setting. The experiment was also stopped when people walked into the setting from the opposite direction (i.e. exiting the city).

In the moderately intense respect condition we used a respect cue similar to the moderately intense condition in Study 2. The (second) confederate (again alternating man or woman) in this condition walked on the same sidewalk as the participants but in the opposite direction. The confederate dropped and picked up the empty soda can when (s)he had neared the participant to about 10 meters. This was roughly at a distance of 20 meters from where the orange” confederate was standing.

The (second) confederate (again alternating man or woman and seemingly a resident) in our third, most intense respect condition was sweeping the pavement at the building side (i.e. far from the roadside). This confederate was positioned at about 20–25 meters from the orange confederate and in such a way that the approaching participant was not obstructed in in his or her walk. The swept litter consisted at this moment of an empty soda can, making the disapproval both visible and audible.

## Supporting Information

Data S1 **Dataset and description of Study 1.**
(PDF)

Data S2 **Dataset and description of Study 2.**
(PDF)

Data S3 **Dataset and description of Study 3.**
(PDF)

Analysis S1 **Analyses of potential differences in prosocial behavior between groups and individuals and between men and women, in Study 2.**
(PDF)

Analysis S2 **Analyses of potential differences in prosocial behavior between men and women and as a result of gender differences between confederate and participant, in Study 3.**
(PDF)
