# Supplementary material for: The Importance of Demonstratively Restoring Order
Source: PLoS One. 2013 Jun 5;8(6):e65137. doi: 10.1371/journal.pone.0065137 (PMC3673976; doi:10.1371/journal.pone.0065137)
Supplement: Data S1 — Dataset and description of Study 1. (PDF) [file pone.0065137.s001.pdf]

Condition:	1 = Subtle disrespect (Garbage bags)<br>2 = Subtle respect (Clean/ No garbage bags)
Prosoc:	0 = No action (Letter is not posted)<br>1 = Prosocial action (Letter is posted)

Condition	Prosoc
1	0
1	0
1	0
1	0
1	0
1	0
1	0
1	0
1	0
1	0
1	0
1	0
1	0
1	0
1	0
1	0
1	0
1	0
1	0
1	0
1	0
1	0
1	0
1	0
1	0
1	0
1	0
1	0
1	0
1	0
1	0
1	0
1	0
1	0
1	0
1	0
1	0
1	0
1	0
1	0
1	0

1	0
1	0
1	0
1	0
1	0
1	0
1	0
1	0
1	0
1	0
1	0
1	0
1	0
1	0
1	0
1	0
1	0
1	0
1	0
1	0
1	0
1	0
1	0
1	0
1	0
1	0
1	0
1	0
1	0
1	0
1	0
1	0
1	0
1	0
1	0
1	0
1	0
1	0
1	0
1	0
1	0
1	0
1	0
1	0
1	0
1	0
1	0
1	0
1	0
1	0
1	0
1	0
1	0

1	0
1	0
1	0
1	0
1	0
1	0
1	0
1	0
1	0
1	0
1	0
1	0
1	0
1	0
1	0
1	0
1	0
1	0
1	0
1	0
1	0
1	0
1	0
1	0
1	0
1	0
1	0
1	0
1	0
1	0
1	0
1	0
1	0
1	0
1	0
1	0
1	0
1	0
1	1
1	1
1	1
1	1
1	1
1	1
1	1
1	1
1	1
1	1
1	1
1	1
1	1
1	1
1	1

2	0
2	0
2	0
2	0
2	0
2	0
2	0
2	0
2	0
2	0
2	0
2	0
2	0
2	0
2	0
2	0
2	0
2	0
2	0
2	0
2	0
2	0
2	0
2	0
2	0
2	0
2	0
2	0
2	0
2	0
2	0
2	0
2	0
2	0
2	0
2	0
2	0
2	0
2	0
2	0
2	0
2	0
2	0
2	0
2	0
2	0
2	0
2	0
2	0
2	0
2	0
2	0
2	0

2	0
2	0
2	0
2	0
2	0
2	0
2	0
2	0
2	0
2	0
2	0
2	0
2	0
2	0
2	0
2	0
2	0
2	0
2	0
2	0
2	0
2	0
2	0
2	0
2	0
2	0
2	0
2	0
2	0
2	0
2	0
2	0
2	0
2	0
2	0
2	0
2	0
2	0
2	0
2	0
2	0
2	0
2	0
2	0
2	0
2	0
2	0
2	0
2	0
2	0
2	1
2	1
2	1

2	1
2	1
2	1
2	1
2	1
2	1
2	1
2	1
2	1
2	1
2	1
2	1
2	1
2	1
2	1
2	1
2	1
2	1
2	1
2	1
2	1
2	1
2	1
2	1
2	1
2	1
2	1
2	1
2	1
2	1
