# Supplementary material for: The Importance of Demonstratively Restoring Order
Source: PLoS One. 2013 Jun 5;8(6):e65137. doi: 10.1371/journal.pone.0065137 (PMC3673976; doi:10.1371/journal.pone.0065137)
Supplement: Data S2 — Dataset and description of Study 2. (PDF) [file pone.0065137.s002.pdf]

Condition:	1 = Subtle disrespect (Garbage bags)<br>2 = Subtle respect (Clean/ No garbage bags)<br>3 = Moderately intense respect (Confederate picks-up can)	
Gender:	1 = (Participant is) Man<br>2 = (Participant is) Woman<br>3 = (Participant is) Group	
Group:	0 = (Participant is) Individual<br>1 = (Participant is) Group	
Groupnumb:	0 = (Participant is) Individual<br>1 = (Participant is) Group = 2 people<br>2 = (Participant is) Group >2 people	
Prosoc:	0 = No action (Bicycle is not picked-up)<br>1 = Prosocial action (Bicycle is picked-up)	

Condition	Gender	Group	Groupnumb	Prosoc
1	1	0	0	0
1	1	0	0	0
1	1	0	0	0
1	1	0	0	0
1	1	0	0	0
1	1	0	0	0
1	1	0	0	0
1	1	0	0	0
1	1	0	0	0
1	1	0	0	0
1	1	0	0	0
1	1	0	0	0
1	1	0	0	0
1	1	0	0	0
1	1	0	0	0
1	1	0	0	0
1	1	0	0	0
1	1	0	0	0
1	1	0	0	0
1	1	0	0	0
1	1	0	0	0
1	1	0	0	0
1	1	0	0	0
1	1	0	0	0
1	1	0	0	0
1	1	0	0	0
1	1	0	0	0
1	1	0	0	0
1	1	0	0	0

1	1	0	0	0
1	1	0	0	0
1	1	0	0	0
1	1	0	0	0
1	1	0	0	0
1	1	0	0	0
1	1	0	0	0
1	1	0	0	0
1	1	0	0	0
1	1	0	0	0
1	1	0	0	0
1	1	0	0	0
1	1	0	0	0
1	1	0	0	0
1	1	0	0	0
1	1	0	0	0
1	1	0	0	0
1	1	0	0	0
1	1	0	0	0
1	1	0	0	0
1	1	0	0	0
1	1	0	0	0
1	1	0	0	0
1	1	0	0	0
1	1	0	0	1
1	1	0	0	1
1	1	0	0	1
1	1	0	0	1
1	2	0	0	0
1	2	0	0	0
1	2	0	0	0
1	2	0	0	0
1	2	0	0	0
1	2	0	0	0
1	2	0	0	0
1	2	0	0	0
1	2	0	0	0
1	2	0	0	0
1	2	0	0	0
1	2	0	0	0
1	2	0	0	0
1	2	0	0	0
1	2	0	0	0
1	2	0	0	0
1	2	0	0	0
1	2	0	0	0
1	2	0	0	0
1	2	0	0	1
1	3	1	1	0
1	3	1	1	0
1	3	1	1	0
1	3	1	1	0
1	3	1	1	0

1	3	1	1	0
1	3	1	1	0
1	3	1	1	0
1	3	1	1	0
1	3	1	1	0
1	3	1	1	0
1	3	1	1	0
1	3	1	1	0
1	3	1	1	0
1	3	1	1	0
1	3	1	1	0
1	3	1	1	0
1	3	1	1	0
1	3	1	1	1
1	3	1	2	0
1	3	1	2	0
2	1	0	0	0
2	1	0	0	0
2	1	0	0	0
2	1	0	0	0
2	1	0	0	0
2	1	0	0	0
2	1	0	0	0
2	1	0	0	0
2	1	0	0	0
2	1	0	0	0
2	1	0	0	0
2	1	0	0	0
2	1	0	0	0
2	1	0	0	0
2	1	0	0	0
2	1	0	0	0
2	1	0	0	0
2	1	0	0	0
2	1	0	0	0
2	1	0	0	0
2	1	0	0	0
2	1	0	0	0
2	1	0	0	0
2	1	0	0	0
2	1	0	0	0
2	1	0	0	0
2	1	0	0	0
2	1	0	0	0
2	1	0	0	0
2	1	0	0	0
2	1	0	0	0
2	1	0	0	0
2	1	0	0	0
2	1	0	0	0
2	1	0	0	0
2	1	0	0	0
2	1	0	0	0

2	1	0	0	0
2	1	0	0	0
2	1	0	0	0
2	1	0	0	0
2	1	0	0	0
2	1	0	0	0
2	1	0	0	0
2	1	0	0	0
2	1	0	0	0
2	1	0	0	0
2	1	0	0	0
2	1	0	0	1
2	1	0	0	1
2	1	0	0	1
2	1	0	0	1
2	1	0	0	1
2	1	0	0	1
2	1	0	0	1
2	1	0	0	1
2	1	0	0	1
2	1	0	0	1
2	1	0	0	1
2	1	0	0	1
2	2	0	0	0
2	2	0	0	0
2	2	0	0	0
2	2	0	0	0
2	2	0	0	0
2	2	0	0	1
2	3	1	1	0
2	3	1	1	0
2	3	1	1	0
2	3	1	1	0
2	3	1	1	0
2	3	1	1	0
2	3	1	1	0
2	3	1	1	0
2	3	1	1	0
2	3	1	1	0
2	3	1	1	0
2	3	1	1	0
2	3	1	1	0
2	3	1	1	0
2	3	1	1	0
2	3	1	1	1
2	3	1	1	1
2	3	1	1	1
2	3	1	1	1
2	3	1	1	1
2	3	1	1	1
2	3	1	2	0
2	3	1	2	0
2	3	1	2	0

2	3	1	2	0
2	3	1	2	1
3	1	0	0	0
3	1	0	0	0
3	1	0	0	0
3	1	0	0	0
3	1	0	0	0
3	1	0	0	0
3	1	0	0	0
3	1	0	0	0
3	1	0	0	0
3	1	0	0	0
3	1	0	0	0
3	1	0	0	0
3	1	0	0	0
3	1	0	0	0
3	1	0	0	0
3	1	0	0	0
3	1	0	0	0
3	1	0	0	0
3	1	0	0	0
3	1	0	0	0
3	1	0	0	0
3	1	0	0	0
3	1	0	0	0
3	1	0	0	1
3	1	0	0	1
3	1	0	0	1
3	1	0	0	1
3	1	0	0	1
3	1	0	0	1
3	1	0	0	1
3	1	0	0	1
3	1	0	0	1
3	1	0	0	1
3	1	0	0	1
3	1	0	0	1
3	1	0	0	1
3	1	0	0	1
3	2	0	0	0
3	2	0	0	0
3	2	0	0	0
3	2	0	0	0
3	2	0	0	0
3	2	0	0	0
3	2	0	0	0
3	2	0	0	0
3	2	0	0	0
3	2	0	0	0
3	2	0	0	0
3	2	0	0	0
3	2	0	0	0
3	2	0	0	0

3	2	0	0	1
3	2	0	0	1
3	2	0	0	1
3	2	0	0	1
3	2	0	0	1
3	3	1	1	0
3	3	1	1	0
3	3	1	1	0
3	3	1	1	0
3	3	1	1	0
3	3	1	1	0
3	3	1	1	0
3	3	1	1	0
3	3	1	1	0
3	3	1	1	0
3	3	1	1	0
3	3	1	1	0
3	3	1	1	0
3	3	1	1	0
3	3	1	1	0
3	3	1	1	0
3	3	1	1	0
3	3	1	1	0
3	3	1	1	0
3	3	1	1	0
3	3	1	1	0
3	3	1	1	0
3	3	1	1	0
3	3	1	1	0
3	3	1	1	0
3	3	1	1	1
3	3	1	1	1
3	3	1	1	1
3	3	1	1	1
3	3	1	1	1
3	3	1	1	1
3	3	1	1	1
3	3	1	1	1
3	3	1	1	1
3	3	1	1	1
3	3	1	1	1
3	3	1	1	1
3	3	1	2	0
3	3	1	2	0
3	3	1	2	0
3	3	1	2	1
3	3	1	2	1
3	3	1	2	1
