# Supplementary material for: The Importance of Demonstratively Restoring Order
Source: PLoS One. 2013 Jun 5;8(6):e65137. doi: 10.1371/journal.pone.0065137 (PMC3673976; doi:10.1371/journal.pone.0065137)
Supplement: Analysis S1 — Analyses of potential differences in prosocial behavior between groups and individuals and between men and women, in Study 2. (PDF) [file pone.0065137.s004.pdf]

Additional analyses Study 2

The percentage of individuals versus the percentage of groups that acted prosocial in the in the various conditions of Study 2:

Condition 1 (subtle disrespect): individual (N=77) 6% vs groups (N=21) 5% (z=.293, p=.769 two-sided)

Condition 2 (subtle respect): individual (N=66) 20% vs groups (N=26) 27% (z=.756, p=.449 two-sided)

Condition 3 (moderate respect): individual (N=56) 34% vs groups (N=43) 35% (z=.100, p=.921 two-sided)

The percentage of men versus the percentage of woman that acted prosocial in the in the various "individual" conditions of Study 2:

Condition 1 (subtle disrespect): men (N=57) 7% vs women (N=20) 5% (z=.315, p=.753 two-sided)

Condition 2 (subtle respect): men (N=60) 20% vs women (N=6) 8% (z=.195, p=.845 two-sided)

Condition 3 (moderate respect): men (N=37) 38% vs women (N=19) 26% (z=.862, p=.389 two-sided)
