# Supplementary material for: The Importance of Demonstratively Restoring Order
Source: PLoS One. 2013 Jun 5;8(6):e65137. doi: 10.1371/journal.pone.0065137 (PMC3673976; doi:10.1371/journal.pone.0065137)
Supplement: Analysis S2 — Analyses of potential differences in prosocial behavior between men and women and as a result of gender differences between confederate and participant, in Study 3. (PDF) [file pone.0065137.s005.pdf]

Additional analyses Study 3

The percentage of men versus the percentage of woman that acted prosocial in the in the various conditions of Study 3:

Condition 1 (subtle respect): men (N=28) 50% vs women (N=22) 27% (z=.1.623, p=.103 two-sided)

Condition 2 (moderate respect): men (N=33) 58% vs women (N=23) 74% (z=1.255, p=.209 two-sided)

Condition 3 (intense respect): men (N=40) 80% vs women (N=21) 86% (z=.551, p=.581 two-sided)

The percentage of observations that acted prosocial in the various conditions of Study 3, comparison of the observations, where confederate and participant differed vs not differed in gender:

Condition 1 (subtle respect): no-gender difference (N=24) 25% vs gender difference (N=26) 54% (z=.2.080, p=.002 one-sided)

Condition 2 (moderate respect): no-gender difference (N=27) 70% vs gender difference (N=29) 59% (z=.917, p=.180 one-sided)

Condition 3 (intense respect): no-gender difference (N=26) 77% vs gender difference (N=35) 86% (z=.883, p=.189 one-sided)
